# Participation in club sport in childhood is associated with mental health in preterm and term born adolescents

**DOI:** 10.1007/s00787-023-02365-8

**Published:** 2024-01-30

**Authors:** Juliane Spiegler, Usama EL-Awad, Nicole Baumann, Sakari Lemola, Dieter Wolke

**Affiliations:** 1 Department of Paediatrics, University Hospital of Würzburg, Josef-Schneider-Strasse 2, 97080 Würzburg, Germany; 2 Department of Psychology, University of Warwick, University Road, Coventry, CV4 7AL UK; 3 Faculty of Psychology and Sports Sciences, University of Bielefeld, P.O. Box 10 01 31, 33501 Bielefeld, Germany; 4 Department of Population Health Sciences, University of Leicester, University Road, Leicester, LE1 7RH UK; 5 Turner Institute for Brain and Mental Health, School of Psychological Sciences, Monash University, Level 5, 18 Innovation Walk, Clayton Campus, Melbourne, VIC 3800 Australia; 6 Division of Mental Health & Wellbeing, University of Warwick, University Road, Coventry, CV4 7AL UK

**Keywords:** Longitudinal studies, Prematurity, Mental health, Activity level, Sport participation

## Abstract

Preterm born individuals have an increased risk for mental health problems. Participation in club sport is associated with better mental health but the causal direction is unclear. It is not known whether this association could also be found in preterm children. Data from term born (*N* = 10,368), late preterm (*N* = 630), and very to moderately preterm born (*N* = 243) children from the Millennium Cohort Study at the ages of 3, 5, 7, 11, and 14 years were used. Longitudinal associations between the parent-reported Strengths and Difficulties questionnaire (SDQ) and club sport participation (days per week) were analysed using multigroup structural equation modelling, adjusting for gender, maternal depression, parental education; motor problems and attrition were controlled for. Multi-group structural equation modelling showed that children with more peer relationship problems, emotional symptoms, conduct problems or hyperactivity-inattention were less likely to participate in club sport at subsequent assessment time points. More days with club sport participation was associated with lower levels of emotional symptoms and peer relationship problems but not conduct problems or hyperactivity-inattention at subsequent ages. Results were similar in all gestational age groups. Club sport participation predicts lower levels of peer relationship and emotional problems in subsequent waves while it is also predicted by lower levels of emotional problems, peer relationship problems, conduct problems and hyperactivity-inattention in preceding waves. Since no differences in the relationship between SDQ subscales and club sport participation were seen with regard to gestational age groups, club sport should be encouraged in all children.

**Supplementary Information:**

The online version contains supplementary material available at 10.1007/s00787-023-02365-8.

## Introduction

Mental health problems are frequent in adolescents and young adults and have been associated with several biological (e.g., birth complications, genetic traits, physical health) and social risk (parental education) factors [28]. Most mental health problems start early in life with a high recurrence risk in adulthood [9, 19]. Half of all lifetime cases of mental health disorders have their onset before the age of 14 years [20]. Within the age group of 5–14 year old children and adolescents mental health problems are among the main causes of disease burden in developed countries [2]. Therefore, it is important to identify protective factors early in life. Ideally these protective factors should be suitable for prevention or intervention.

Physical activity has been associated with better mental health [36]. In the Millennium Cohort Study, higher objectively measured physical activity at the age of 7 years was associated with fewer peer relationship problems at the age of 11 years; however, in the same study higher physical activity was associated with more hyperactivity and conduct problems [1]. Therefore, the benefit of physical activity might not be solely the physical activity as such but depend on the type of physical activity as well. Investigations of the association between club sport participation (CSP) and mental health problems have been limited to cross-sectional and retrospective studies [11, 15, 27]. Few longitudinal studies tried to address whether mental health predicts participation in club sport or vice-versa. In an Australian population-based sample [40], higher scores of the Strengths and Difficulties Questionnaire (SDQ [13]) for both internalizing (i.e., peer relationship, emotional symptoms) and externalizing problems (i.e., conduct, hyperactivity) that indicated more problems at the age of 12 years were associated with less CSP at the age of 14 years. Conversely, higher CSP at age 12 was associated with lower internalizing problems at the age of 14 years. While the authors showed a bidirectional relationship between SDQ subscales for internalizing problems, CSP was not associated with subsequent externalizing problems [40]. In a further follow-up at the age of 16 years the same study group used cross-lagged panel models and showed that only emotional symptoms were bidirectionally associated with organized team sport but not with organized individual sport [14]. A Canadian study showed positive effects of high participation in team sport between age 6–10 years on depression, anxiety and social withdrawal [42]. A Dutch study that included children between the ages of 8–12 years similarly showed that higher CSP was associated with subsequent lower internalizing problems, but no association was found for externalizing problems [25].

It has been argued that CSP is beneficial for child or adolescent mental health. Children and adolescents engage in club sport to have fun and enjoy their own learning and development [16]. Club sports provide a structure in young people’s lives as well as a meaningful context to meet and engage with friends [16] and improve relationship with peers as well as emotional regulation. Young people are required to accept a system of principles, rules, and requirements for the type of sport they are involved in [23]. Mastering these tasks can improve self-esteem and competence [10]. Theories trying to explain why CSP might improve oppositional behaviour argue that learning to obey rules and authority, learning skills to cope with others, conflict resolution and cooperation with others as well as the opportunity to form social bonds are important for a positive effect of club sport on behaviour [37]. Peer competition can further motivate to improve competence in certain tasks [16] while on the other hand it may also have negative consequences on children’s mental health due to burnout, bitterness and jealousy [3]. Taking all positive and negative aspects of CSP into account, CSP is often recommended by health care professionals for multiple reasons such as improving/maintaining physical or mental health and social interaction [43].

Preterm born children and adolescents are at an increased risk for mental health problems in addition to neuro-cognitive and physical health problems [5, 7, 30, 33]. CSP might be particularly beneficial for preterm children and adolescents. First, it has been shown that cardiorespiratory function is lower in preterm born adolescents and can be improved by sport with similar effect sizes as seen in term born populations [8], which might positively affect their wellbeing and mental health. Second, preterm children more often show socially withdrawn behaviour associated with shyness, peer problems, and social anxiety [17, 31]. Positive effects of CSP on peer relationships or emotional function seen in term born population [14] might present a cost-effective, easy accessible intervention for preterm born children and adolescents who are at an increased risk to develop these problems [5, 7]. In a population-based cohort from the UK and a study of preterm born from Switzerland higher levels of physical activity measured by accelerometer in a cross-sectional design were associated with lower peer relationship problems in adolescence [6] and these associations were comparable in term born and preterm born groups. Overall, prospectively collected data on the potential benefit of CSP on later mental health are scarce and are, to the best of our knowledge, completely missing for children born preterm.

The aim of the current study was to investigate temporal associations of CSP (age 5–11 years) and aspects of mental health such as peer relationship, emotional problems, conduct problems or hyperactivity/inattention (3–14 years) and to examine whether temporal associations between CSP and mental health scores differ in groups defined by gestational age. Our hypothesis was that the association of CSP and mental health at a later age in more immature gestational age groups differs to those effects described in term born populations.

## Methods

The Millennium Cohort Study (MCS) is a representative longitudinal study of 18,818 infants born between September 2000 and January 2002 in the United Kingdom [26]. A random sample was drawn from Child Benefit registers using over-sampling of ethnic minority and disadvantaged areas. Parents were interviewed for the first time when their children were aged 9 months (survey 1) and again at the age of 3 (survey 2), 5 (survey 3), 7 (survey 4), 11 (survey 5), 14 (survey 6) and 17 (survey 7) years. Of the initial sample 61% participated in the 6th survey. The study design allowed to control for attrition. Detailed information was collected on maternal, socio-economic, and child related factors. Ethical approval and written informed consent were obtained for all surveys (London—Hampstead Research Ethics Committee, REC reference 14/LO/0868). Preterm born children are represented in the expected frequency for the UK[21]; there were no exclusion criteria apart from missing parental consent, especially none of the children were excluded due to physical or mental health problems.

### Outcome variables

Primary caregivers were asked to assess their children’s mental health using the Strengths and Difficulties Questionnaire (SDQ [13]) at the age of 3, 5, 7, 11, 14 and 17 years. The SDQ consist of 25 items comprising five subscales measuring emotional symptoms, conduct problems, hyperactivity-inattention, peer relationship problems, and prosocial behaviour. Each item is scored on a 3-point Likert-type scale: 0 = not true, 1 = somewhat true, and 2 = certainly true; 20 items excluding the subscale on prosocial behaviour are used to calculate the total problem score (range 0–40) with higher scores indicating more problems.

### Gestational age

Gestational age in weeks was calculated using the mother’s report of the expected due date, which corresponded well with data in routine hospital records [29]. Gestational age was grouped into three categories: 1 = term (37 + 0 weeks of gestation and above), 2 = late preterm (34 + 0 to 36 + 6 weeks of gestation), 3 = moderately to very preterm (< 34 weeks of gestation). The small group size in relation to the number of control variables made further differentiation of preterm born groups impossible.

### Club sport participation (CSP)

At the age of 5, 7, and 11 years parents reported on the frequency on average of their child’s physical activity in a club or class ranging from 0 to 5 days per week. Detailed frequencies of CSP in the different gestational age groups have been published before [35].

### Control variables

*Parental education* (highest education of the child’s parents or carer classified as high (equivalent to high school diploma) or lower) was used as a proxy for family socio-economic status as in a previous analysis of this dataset parental education showed a significant association with physical activity after controlling for a variety of socio-economic factors[35]. *Maternal depression* (doctor diagnosed yes/no) was inquired during the first survey.

*Severe motor problems* inquired by parental interview were defined as being diagnosed with a longstanding health condition according to International Classification of Diseases codes that implicate motor problems at the age of 5 years (G80-G83 [cerebral palsy and other paralytic syndromes], R26-R29 [abnormalities of gait and mobility, other lack of coordination, other symptoms and signs involving the nervous and musculoskeletal systems] Q05 [Spina bifida], G14 [post-polio syndrome]).

### Statistics

SPSS version 27 (IBM, Armonk, New York) was used for descriptive data analyses. R 3.6.3 (R Core Team, 2021) and its associated packages foreign (v0.8–81; R Core Team, 2020), lavaan (v0.6–8; Rosseel, 2021), survey (v4.0; Lumley, 2020) and lavaan.survey (v1.1.3.1; Oberski, 2016; Oberski, 2014) were used for data preparation and the structural equation model (SEM) analyses. All data were controlled for attrition by using complex sample analysis with the non-response weights provided by the study centre of the MCS [18].

To determine whether preterm born groups in the MCS show higher rates of mental health problems, mean scores of the SDQ subscales for prosocial behaviour, peer relationship problems, emotional symptoms, hyperactivity-inattention and conduct problems were compared across gestational age groups. Mean scores of the gestational age groups were adjusted in the general linear model for known confounders (gender [2], parental education [39], maternal depression [32], motor problems [35]). Means and 95% confidence intervals for the different gestational age groups were analysed at each age separately. Means of the five subscales and SDQ total score for very to moderate and late preterm were compared to term born children using *t* test. A *p* value of < 0.05 (Bonferroni adjusted) was regarded as statistically significant.

A multigroup cross-lagged panel model was estimated in R with the lavaan package to examine the longitudinal association between the four SDQ subscales of peer relationship problems, emotional symptoms, hyperactivity-inattention and conduct problems (df = 102) on subsequent participation in CSP and vice-versa controlling for autoregressions. In detail, the models were fitted as a Multigroup SEM with the lavaan package, comparing means of days with CSP at different ages and means of the four SDQ subscales between the very to moderately preterm, late preterm, and full term group, using full information maximum likelihood (FIML) estimation, while accounting for the control variables (parental education, maternal depression, gender). Here, the cross-lagged paths were allowed to be estimated freely between the groups, with the other paths fixed. In order to consider the complex survey design (clusters, attrition/ non-response weights, strata and finite population correction), the lavaan objects were re-fitted with the lavaan survey package while using the maximum likelihood approach with robust standard errors (adjusted for mean and variance, based on the Satterthwaite approach; see 34). Finally, the parameters of the cross-lagged paths of both preterm groups were each compared pairwise (*df* = 1) with the corresponding cross-lagged paths of the full term group using the Wald *χ*^2^ test statistic, whereby the Benjamini and Hochberg procedure [4] was applied to decrease the false discovery rate (FDR).

A sensitivity analysis was conducted through the comparison of equivalent models with the same number of degrees of freedom (*df* = 102) following the structure of models and consisting of participants with and without severe motor impairments as defined above. To avoid bias in the weighting procedure, a data subset containing solely participants without severe motor impairments was subsequently formed based on the survey design object referring to the total sample.

## Results

A flow chart of study participation at the different ages can be found in the Supplement (Fig. S1). Demographic characteristics of the whole cohort and those with longitudinal data for CSP at the age of 5, 7, and 11 years are shown in Table S1. Higher educated families were more likely to continue participation in the MCS.

There were significant differences between gestational age groups for emotional symptoms, peer relationship problems and hyperactivity-inattention (Fig S2, Table S2). More immature born groups had significantly higher scores for emotional symptoms at the ages of 3, 5, 11, 14 and 17 years (*p* < 0.05), for peer relationship problems at 3, 5, 14 and 17 years (*p* < 0.05), for hyperactivity at 3, 5, 7, and 11 years (*p* < 0.05), and for conduct problems higher scores were only seen at age 11 years (*p* < 0.05). However, there were no group differences for prosocial behaviour.

**Fig. 1:**
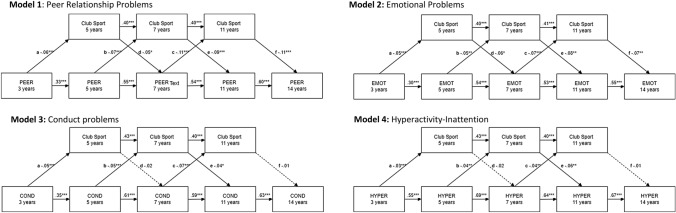
Cross lagged panel modelling of the longitudinal relationship between club sport participation with a minimum of 0 and a maximum of 5 days per week of club sport participation at a certain age and parental rated Strength and Difficulties Questionnaire subscales with a minimum of 0 and maximum of 10 point at a certain age is shown including all gestational age groups. Multigroup SEM based on cross-lagged panel design with very to moderate preterm (*n* = 220), late preterm (*n* = 585), and term born (*n* = 10,371) showed no differences between gestational age groups. Model 1: CFI = 0.90, TFI = 0.91, RMSM = 0.04; Model 2: CFI = 0.93, TFI = 0.91, RMSM = 0.04; Model 3: CFI = 0.87, TFI = 0.88, RMSM = 0.05; Model 4: CFI = 0.95, TFI = 0.93, RMSM = 0.04. Continuous line indicate significant path, dotted line indicate non-significant path. Note. *p < 0.05. **p < 0.01. ***p < 0.001. Standardized regression weights are shown

For all cross-lagged panel models higher problem scores were associated with lower CSP rates at subsequent ages with small effect sizes (Fig. 1). Higher CSP was associated with fewer peer relationship problems and emotional symptoms at later age with a small effect size. For example, one more day of CSP at age 11 (CSP at age 11 ranging from 1.7 to 1.9 days per weeks with a SD of 1.5 to 1.6 across gestational ages) decreased the mean score of peer relationship problems by 0.7 points at the age of 14 years (SDQ at age 14 years ranging from 8.1 to 9.2 with a SD of 5.9–6.2 across gestational ages). However, associations between CSP and subsequent conduct problems and hyperactivity-inattention were consistently not significant across ages.

The multigroup analysis indicated no differences in the relationship between CSP and SDQ subscales across gestational age groups (Table 1).

**Table 1:** Pairwise comparisons of cross-lagged paths between gestational age groups

	Peer relationship problems (Model 1)		Emotional problems (Model 2)		Conduct problems (Model 3)		Hyperactivity-inattention (Model 4)	
Parameter constraints	Wald *χ*^2^ (*df* = 1)	^***^p (*p*_FDR_)	Wald *χ*^2^ (*df* = 1)	^***^p (*p*_FDR_)	Wald *χ*^2^ (*df* = 1)	*p* (*p*_FDR_)	Wald *χ*^2^ (*df* = 1)	*p* (*p*_FDR_)
SDQ subscale at age 3 years → Club sport participation at age 5 years (path “a”)								
Very to moderately preterm vs. term born	^**^4.61	0.032 (0.384)	^**^0.32	0.571 (0.883)	0.82	0.364 (0.411)	0.68	0.408 (0.612)
Late preterm vs. term born	^**^0.17	0.684 (0.919)	^**^3.97	0.046 (0.306)	1.30	0.254 (0.381)	2.20	0.138 (0.276)
SDQ subscale at age 5 years → Club sport participation at age 7 years (path “b”)								
Very to moderately preterm vs. term born	< 0.01	0.949 (0.998)	^**^0.30	0.585 (0.883)	1.82	0.177 (0.303)	2.74	0.098 (0.235)
Late preterm vs. term born	^**^0.09	0.766 (0.919)	^**^0.03	0.871 (0.883)	1.06	0.304 (0.405)	4.90	0.027 (0.152)
SDQ subscale at age 7 years → Club sport participation at age 11 years (path “c”)								
Very to moderately preterm vs. term born	< 0.01	0.998 (0.998)	^**^0.15	0.702 (0.883)	2.17	0.141 (0.282)	0.29	0.590 (0.708)
Late preterm vs. term born	^**^0.09	0.763 (0.919)	^**^0.02	0.883 (0.883)	0.78	0.377 (0.411)	4.31	0.038 (0.152)
Club sport participation at age 5 years → SDQ subscale at age 7 years (path “d”)								
Very to moderately preterm vs. term born	^**^0.19	0.666 (0.919)	^**^0.04	0.833 (0.883)	0.67	0.413 (0.413)	0.33	0.568 (0.708)
Late preterm vs. term born	^**^1.52	0.218 (0.654)	^**^1.35	0.245 (0.883)	5.24	0.022 (0.168)	0.10	0.750 (0.796)
Club sport participation at age 7 years → SDQ subscale at age 11 years (path “e”)								
Very to moderately preterm vs. term born	^**^2.27	0.132 (0.528)	^**^0.66	0.418 (0.883)	2.22	0.136 (0.282)	3.44	0.063 (0.189)
Late preterm vs. term born	^**^0.11	0.737 (0.919)	^**^0.25	0.620 (0.883)	2.35	0.126 (0.282)	1.10	0.293 (0.502)
Club sport participation at age 11 years → SDQ subscale at age 14 years (path “f”)								
Very to moderately preterm vs. term born	^**^2.44	0.118 (0.528)	^**^0.22	0.640 (0.883)	3.39	0.066 (0.264)	0.07	0.796 (0.796)
Late preterm vs. term born	^**^1.08	0.299 (0.718)	^**^3.80	0.051 (0.306)	4.84	0.028 (0.168)	8.97	0.003 (0.036)

See Fig. 1 for the assignment of parameter constraints

Multigroup SEM with very to moderate preterm, *n* = 220; late preterm, n = 585; full term born, *n* = 10,371 did not detect significant differences between the different gestational age groups in the four different models of SDQ subscales analysed. Data are shown for the different paths comparing different gestational age groups. Even though single paths might show significant difference the analysis of four different whole models did not detect differences between the gestational age groups, i.e., the effects shown in the Multigroup SEM in Fig. 1 applies to all children regardless of their differences in gestational age; Model 1: *CFI* = 0.90, *TLI* = 0.91, *SRMR* = 0.04; Model 2: *CFI* = 0.93, *TLI* = 0.91, *SRMR* = 0.04; Model 3: *CFI* = 0.87, *TLI* = 0.88, *SRMR* = 0.05; Model 4: *CFI* = 0.95, *TLI* = 0.93, *SRMR* = 0.04. All models are controlled for gender, parental education, maternal depression and attrition using complex sample analysis

In the sensitivity analysis, the change in the fit values and effect sizes for models including children with and without severe motor problems indicated no differences between the models with ∆CFI, ∆TLI, and ∆SRMR being smaller or equal to 0.001 in all analysed models. Therefore, the association between CSP and mental health remained the same in the sensitivity analysis as in the main SEM models.

## Discussion

Results from this longitudinal, population-based cohort from the United Kingdom show that children with more emotional symptoms, peer relationship problems, conduct problems, and hyperactivity-inattention problems participated less frequently in CSP at a later age. However, underlining the importance of club sport, more days per week of CSP was predictive of decreases in emotional symptoms and peer relationship problems at a later age, while this was not the case for hyperactivity-inattention or conduct problems. The association of lower CSP and mental health problems has been described in several cross-sectional studies [11, 27]. Having lower mental health seems to be a barrier to subsequent CSP and missing out on positive achievement goals and social interaction through CSP. In turn, missing out on CSP increases the risk for lower mental health. Thus, policies that support access to club sport that is attractive to children who are at risk for mental health problems as an avenue to reduce overall mental health burden are needed. Future studies should analyse possible barriers for children with lower mental health, identify types of club sport that are suitable for certain mental health problems or analyse which kind of special support might be helpful to ensure participation in club sport for all children and those with mental health problems in particular.

Taking part in club sport is associated with higher levels of physical activity [41]. We were interested in the potential other benefits of club sport. Apart from increasing physical activity, CSP includes meeting peers with a common interest, a structured training and trainers [3]. Objectively measured physical activity in the MCS at the age of 7 years did not predict better mental health at the age of 11 years [1]. However, our study shows that CSP between the ages of 5 to 11 years has a potentially protective effect for emotional and peer relationship problems. Similar bidirectional effects of organized sport and internalizing problems have been shown in an Australian [14, 40] and a Dutch cohort [25]. While those two studies analysed data over a time period of two to four years during childhood and early adolescence, the current study covers a broader age range from 5 to 11 years for CSP and from age 3 to 14 years for SDQ scores. It provides further evidence of a bidirectional effect for emotional symptoms and peer relationship problems and CSP from an early age onward. Thus, the positive association of more CSP and lower mental health problems apply similarly in early childhood as in adolescence. Therefore, club sport should be encouraged from an early age onward and at any age subsequently. Further prospective studies assessing participation in youth groups as well as both CSP and objectively measured physical activity are needed to disentangle whether the physical activity as such positively influences mental health or rather the structured contact to peers that might be achieved by different types of youth groups as well. Whatever the specific mechanisms, encouraging CSP should be in the repertoire of social prescribing by any health professional.

In the current study, no association between CPS and conduct problem or hyperactivity-inattention were found. Similar results have been reported elsewhere [14, 25, 40]. Some intervention studies reported a positive association of club sport on externalizing problems, especially delinquency [37]. However, the positive effect depended on context factors like sociomoral climate and motivation behaviour of the coach in the sport club. These specific variables were not available in the present study nor in any of the observational, longitudinal studies reviewed above [14, 25, 40].

In the present study preterm born children and adolescents showed more mental health problems, i.e., emotional symptoms, peer relationship problems and hyperactivity-inattention compared to term born participants, while conduct problems were comparable across all gestational age groups. This finding is in line with the existing literature [12, 22, 24, 38]. In contrast, the association of physical activity on mental health in preterm born children and adolescence has rarely been studied. Previous cross-sectional analysis of the association of mental health and objectively measured physical activity found no difference between those born preterm and at term [6]. The current Multigroup SEM showed similar effects for all gestational age groups, thus we had to discard our hypothesis that the association of CSP and SDQ subscales differs in the preterm born population. Lower mental health is a comparable barrier to CSP across the gestational range and preterm born children do benefit as much from CSP regarding their mental health as term born peers. Very to moderate preterm born children scored about 0.3–0.5 points higher on emotional and peer relationship symptoms compared to term born peers. Just taking part 1 day per week more in CSP was associated with a reduction of about 0.7 points on emotional or peer relationship problems at subsequent ages. Just 1 day higher CSP could therefore reduce the emotional and peer relationship difficulties in preterm born children compared to term children. Thus, CPS should be actively encouraged for preterm children and their families in follow-up clinics and by primary care health professionals.

### Strength and limitations

The large sample size of the current study allowed controlling for several potential confounding factors including attrition by complex sample design. However, despite adequate statistical power, we were not able to analyse the possible associations in those born very (less than 32 weeks of gestation) or extremely (less than 28 weeks of gestation) preterm due to the low numbers in that range of gestational age. Due to the smaller groups size of preterm born groups we could only have detected large differences in the association between CSP and SDQ subscales compared to term born peers; to detect minor differences this analysis should be repeated in larger preterm born cohorts. While the medical diagnosis of severe motor problems at the age of 5 years was inquired from the parents and included in the sensitivity analysis, we had no information on health problems that are associated with preterm birth, for example bronchopulmonary dysplasia that might affect participation in club sport as well as mental health. We had no information about participation in team vs. individual sport, the type of club sport the children attended and whether children continuously participated in certain types of club sport. Parental report of the CSP “on average of their child per week” might not fully represent the participation over the course of a year. We controlled for parental education as proxy for socio-economic influences on the relationship between CSP and SDQ subscales. Even though this variable was the most predictive in a previous analysis it might not cover all socio-economic influences.

## Conclusion

Even though only a small portion of variance of SDQ subscale scores was explained by CSP, CSP should be encouraged from an early age onwards for the possible beneficial effects on peer relation and emotional symptoms as well as the health benefits. It should be part of social prescribing. However, further studies are necessary to understand which aspects of CSP might positively influence which aspects of mental health. Due to the higher rate of reported mental health problems, preterm born groups could especially profit from intervention studies on the different aspects of CSP with regard to mental health.

### Supplementary Information

Below is the link to the electronic supplementary material.

Supplementary file1 (PDF 489 KB)

## Data Availability

The raw dataset is made available to researchers via the UK Data Service (Centre of Longitudinal Studies; MCS - http://discover.ukdataservice.ac.uk/series/?sn=2000031). Data and code are available 10.1007/s00787-023-02365-8 from the corresponding author upon reasonable request.

## Availability of data

Data were provided by The Centre for Longitudinal Studies, UCL Institute of Education by the UK Data Archive and UK Data Service.

## Code availability

Codes are available on personal request from the authors.
