# Supplementary material for: Participation in club sport in childhood is associated with mental health in preterm and term born adolescents
Source: Eur Child Adolesc Psychiatry. 2024 Jan 30;33(8):2823–30. doi: 10.1007/s00787-023-02365-8 (PMC11272685; doi:10.1007/s00787-023-02365-8)
Supplement: Supplementary file 1 — Supplementary file1 (PDF 489 KB) [file 787_2023_2365_MOESM1_ESM.pdf]

Table S1: Demographic characteristics of the sample stratified for gestational ages and those with longitudinal data about club sport participation

	MCS				With longitudinal club sport data			
	<34 week<br>N= 416	34-36 weeks<br>N=1086	>36 weeks<br>N=17063	p	<34 weeks<br>N=243	34-36 weeks<br>N=630	>37 weeks<br>N=10368	p
Gestational age (weeks)	31.0 (2)	35.6 (1)	39.9 (1)	AB	31.1 (2)	35.6 (1)	39.9 (1)	AB
very preterm	216 (51.9%)	-	-		115 (47.3%)			
Birth weight (gram)	1.69 (0.7)	2.59 (0.5)	3.42 (0.5)	AB	1.69 (0.6)	2.59 (0.5)	3.44 (0.5)	AB
Gender male	225 (54%)	579 (53%)	8703 (51%)	0.17	117 (48.1%)	330 (52.4%)	5151 (49.7%)	0.37
Twins	40 (9.6%)	87 (8.0%)	126 (0.7%)	<0.001	27 (11.2%)	44 (7.0)	74 (0.7%)	<0.001
Triplets	3 (0.7%)	5 (0.5%)	2 (0.0%)		3 (1.2%)	2 (0.3%)	1 (0.0%)	
Ethnicity white	331 (79.8%)	903 (83.1%)	14140 (83.0%)	0.22	201 (82.7%)	534 (84.8%)	8859 (85.6%)	0.47
Maternal depression	139 (33.4%)	318 (29.3%)	4107 (24.1%)	<0.001	77 (31.7%)	182 (28.9%)	2481 (23.9%)	<0.01
High parental education	174 (42.1%)	417 (39.0%)	7268 (43.6%)	<0.01	119 (49.4%)	280 (45.0%)	5135 (50.4%)	0.07
Cognitive delay at age 3								
autism								
Severe motor impairment	11 (3.6%)	6 (0.7%)	31 (0.2%)	<0.001	7 (3%)	4 (0.7%)	22 (0.2%)	<0.001
5 years	0.7 (1.0)	0.9 (1.1)	0.9 (1.1)	A	0.8 (1.0)	0.9 (1.1)	1.0 (1.1)	
7 years	1.3 (1.3)	1.3 (1.3)	1.4 (1.3)		1.3 (1.3)	1.3 (1.3)	1.4 (1.3)	

Club sport participation in days per week	11 years	1.6 (1.5)	1.7 (1.5)	1.9 (1.6)	A	1.7 (1.6)	1.8 (1.5)	1.9 (1.6)	A

Continuous variables are given as mean (standard deviation), categorial variables as absolute numbers and percentage. P values <0.05 for continuous variables with full term as reference coded as comparison to A (very to moderately preterm) B (late preterm)

Table S2: Strength and Difficulties Questionnaire subscales for different gestational age groups

		3 years	5 years	7 years	11 years	14 years	17 years
Peer relationship problems	<34 weeks	1.9 (1.6-2.1)**	1.4 (1.2-1.7)**	1.4 (1.1-1.6)	1.6 (1.3-1.9)	2.3 (2.0-2.6)**	2.3 (1.8-2.8)*
	34-36 weeks	1.5 (1.3-1.6)	1.2 (1.1-1.4)**	1.3 (1.2-1.5)	1.6 (1.4-1.8)	2.1 (1.8-2.3)*	1.7 (1.3-1.8)
	>36 weeks	1.5 (1.4-1.5)	1.1 (1.0-1.1)	1.2 (1.2-1.2)	1.4 (1.3-1.4)	1.8 (1.7-1.8)	1.7 (1.7-1.8)
Emotional symptoms	<34 weeks	1.6 (1.4-1.9)**	1.6 (1.4-1.8)*	1.7 (1.4-2.1)	2.4 (2.0-2.8)**	2.4 (2.0-2.8)	2.5 (2.0-3.0)*
	34-36 weeks	1.5 (1.3-1.6)*	1.5 (1.4-1.6)*	1.6 (1.5-1.8)	2.1 (1.8-2.4)	2.4 (2.1-2.7)*	1.9 (1.3-2.5)
	>36 weeks	1.3 (1.3-1.3)	1.3 (1.3-1.4)	1.5 (1.5-1.6)	1.9 (1.8-1.9)	2.1 (2.0-2.1)	2.0 (1.9-2.0)
Conduct problems	<34 weeks	2.8 (2.6-3.1)	1.6 (1.4-1.8)	1.5 (1.3-1.7)	1.8 (1.5-2.1)*	1.8 (1.4-2.2)	1.1 (0.9-1.4)
	34-36 weeks	2.8 (2.6-2.9)	1.4 (1.3-1.5)	1.4 (1.3-1.6)	1.6 (1.3-1.8)	1.6 (1.4-1.8)	1.1 (0.9-1.3)
	>36 weeks	2.8 (2.7-2.8)	1.5 (1.5-1.5)	1.4 (1.4-1.4)	1.4 (1.4-1.5)	1.5 (1.5-1.6)	1.1 (1.1-1.2)
Hyperactivity-inattention	<34 weeks	4.4 (4.1-4.8)**	3.8 (3.5-4.1)**	3.8 (3.5-4.2)*	3.5 (3.2-3.9)*	3.4 (3.0-3.8)	2.8 (2.3-3.2)
	34-36 weeks	3.9 (3.8-4.2)	3.5 (3.2-3.5)	3.6 (3.4-3.8)*	3.5 (3.2-3.9)*	3.5 (3.2-3.8)*	2.8 (2.4-3.2)
	>36 weeks	3.8 (3.8-3.9)	3.3 (3.2-3.3)	3.4 (3.3-3.4)	3.2 (3.1-3.2)	3.1 (3.1-3.2)	2.4 (2.4-2.5)
Total score	<34 weeks	10.7 (10.0-11.5)***	8.4 (7.8-9.0)***	8.4 (7.7-9.1)*	9.3 (8.4-10.3)**	9.9 (8.7-11.1)*	8.7 (7.3-10.1)*
	34-36 weeks	9.5 (9.0-10.0)	7.5 (7.1-7.9)	8.0 (7.5-8.5)*	8.8 (7.9-9.7)*	9.6 (8.8-10.3)**	7.5 (6.6-8.4)
	>36 weeks	9.4 (9.2-9.5)	7.1 (7.0-7.3)	7.5 (7.3-7.6)	7.9 (7.7-8.0)	8.5 (8.3-8.7)	7.3 (7.1-7.4)
Prosocial	<34 weeks	7.2 (7.0-7.5)	8.4 (8.2-8.7)	8.7 (8.5-8.9)	8.7 (8.4-9.0)	8.1 (7.8-8.5)	8.3 (7.8-8.7)

34-36 weeks	7.4 (7.2-7.5)	8.5 (8.3-8.6)	8.5 (8.3-8.7)	8.7 (8.5-8.9)	8.2 (8.0-8.4)	8.5 (8.2-8.8)
>36 weeks	7.3 (7.3-7.4)	8.4 (8.4-8.4)	8.6 (8.5-8.6)	8.7 (8.7-8.8)	8.2 (8.2-8.3)	8.4 (8.3-8.4)

Mean and 95% confidence interval are adjusted for attrition by complex sample design as well as gender, parental education, maternal depression and motor problems. Gestational age groups are given for very to moderate preterm born (<34 weeks), for late preterm born (34-36 weeks) and term born (>36 weeks). p-values for the comparison of <34 weeks to >36 weeks and 34-36 to >36 weeks are Bonferroni adjusted. *Note* *p<0.05, **p<0.01, *** p<0.001

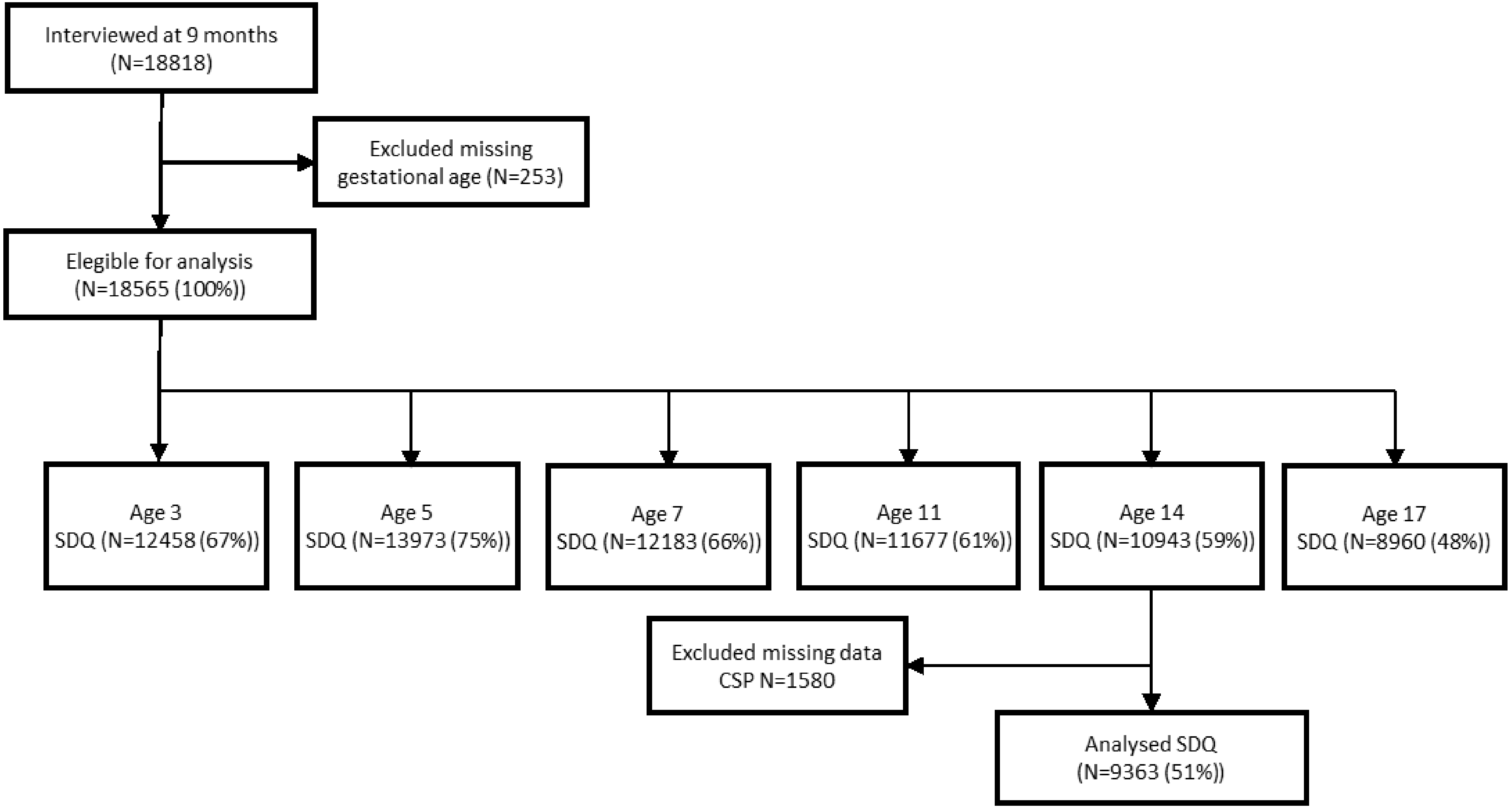

FigS1: Flow chart of study participation and exclusions. Percentages are of the original sample at 9 months with valid gestational age (N=18 565). CSP: club sport participation age 5 to 11. SDQ: strength and difficulties questionnaire

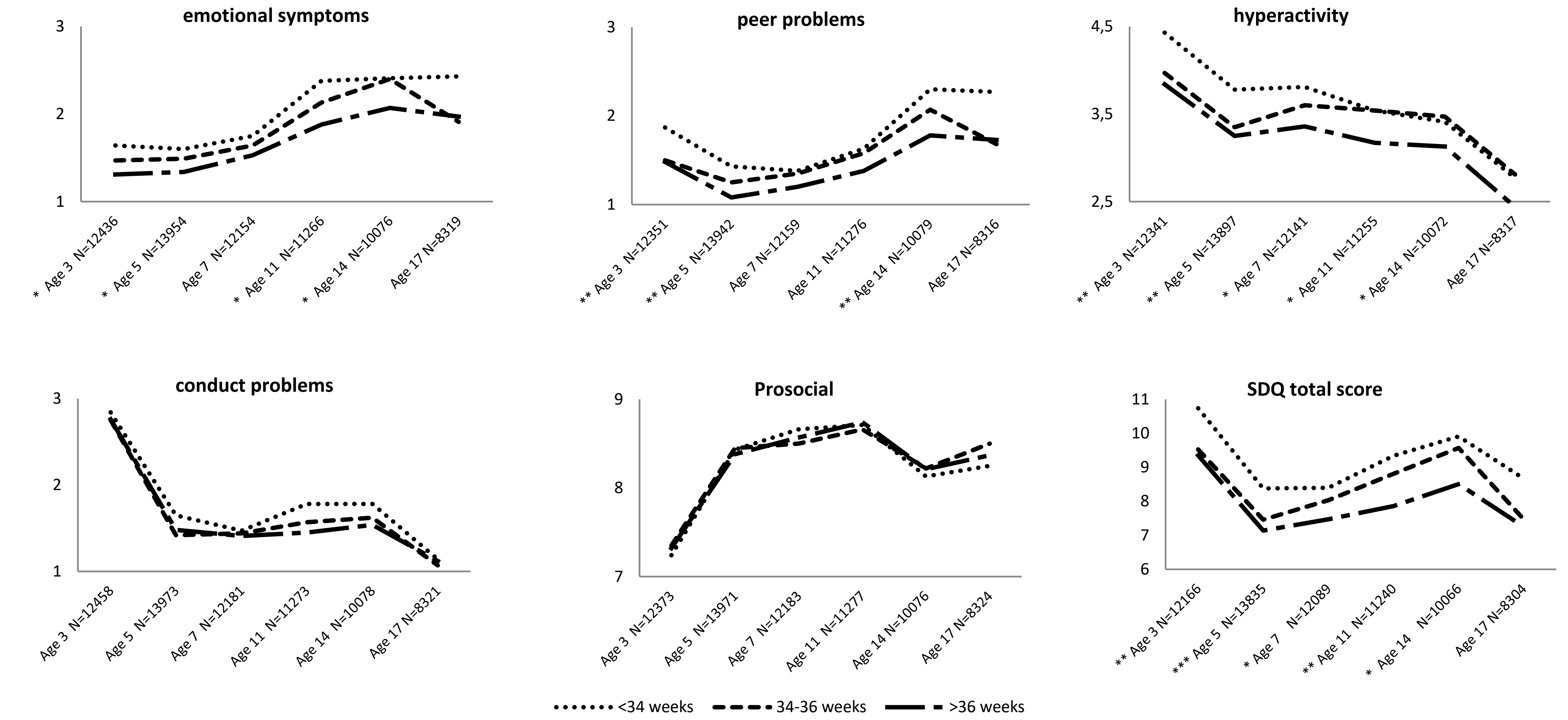

FigS2. Mean parent reported SDQ total score and subscales from the age of 3 to 17 years for different gestational age groups after controlling for attrition (complex sample analysis), gender, maternal depression, parental education (equivalent to high school degree or lower) and motor problems. *p<0.05, **p<0.01, *** p<0.001. All p-values are adjusted (Bonferroni) for multiple testing.
